# Characterization of the Alfalfa Pollen Virome

**DOI:** 10.3390/v18040408

**Published:** 2026-03-25

**Authors:** Lev G. Nemchinov, Sam Grinstead, Olga A. Postnikova, Brian M. Irish

**Affiliations:** 1 Molecular Plant Pathology Laboratory, Beltsville Agricultural Research Center, Agricultural Research Service, U.S. Department of Agriculture, Beltsville, MD 20705, USA; sam.grinstead@usda.gov; 2 Animal Biosciences and Biotechnology Laboratory, Beltsville Agricultural Research Center, Agricultural Research Service, U.S. Department of Agriculture, Beltsville, MD 20705, USA; olga.postnikova@usda.gov; 3 Plant Germplasm Introduction and Testing Research Unit, Agricultural Research Service, U.S. Department of Agriculture, Prosser, WA 99350, USA; brian.irish@usda.gov

**Keywords:** alfalfa (*Medicago sativa* L.), viruses, vertical transmission, pollen transmission

## Abstract

Vertical transmission of plant pathogenic viruses is an important component of viral persistence, survival, and spread in agricultural production systems. This type of transmission is of considerable economic significance as it can cause major crop losses by serving as the initial focus of infection for future epidemics. Vertical transmission occurs when a virus is passed on to offspring either by direct invasion of the developing seed embryo from infected mother plants or through infected pollen grains after fertilization. We have recently demonstrated via high-throughput sequencing that mature seeds of the agriculturally important forage crop alfalfa (*Medicago sativa* L.) are associated with a broad range of viruses, some of which could potentially spread over long distances via seed. With the exception of the alfalfa mosaic virus, little is currently known about viral transmission through alfalfa pollen and its subsequent impact on the disease epidemiology of the crop. The objective of this study was to screen pollen from diverse alfalfa genotypes for pathogenic viruses and assess their risk of transmission. The pollen was collected from alfalfa genotypes selected for fungal disease resistance and agronomic performance in the USDA ARS pre-breeding program in Prosser, WA.

## 1. Introduction

Pollen transmission of plant viruses is a part of the vertical transmission from parents to offspring. It can occur when viral particles in the pollen grains infect the ovule during fertilization, resulting in an infected embryo, seeds, and eventually virus-infected seedlings and mature plants [1,2,3,4]. While most pathogenic viruses are excluded from apical meristems, vertical transmission to the host progeny via invasion of the meristematic tissues and subsequently plant reproductive organs can still take place [5,6]. As pollen is known to travel long distances via airborne particles or insect pollinators [7,8], pollen-associated viruses can achieve rapid geographic expansion and establish themselves in previously unaffected regions.

The dissemination of viruses through pollen is common and has been reported in several agricultural crops [9,10]. However, the current experimental knowledge on pollen transmission of plant viruses in the agriculturally important forage crop alfalfa (*Medicago sativa* L.) is limited to alfalfa mosaic virus (AMV) [11,12]. In greenhouse experiments, Frosheiser [11] showed that the frequency of AMV transmission to alfalfa seeds through pollen ranged from 0.5 to 26.5%.

The first virome of alfalfa seeds recently obtained via high-throughput sequencing (HTS) revealed the presence of sequencing reads corresponding to 27 viruses across 10 different families [13]. The prevailing species were AMV, ubiquitous partiti, amalga and similarly persistent Snake River alfalfa virus (SRAV). Several other viruses not previously associated with alfalfa seeds were also identified [13].

Currently, the specific nature and underlying mechanisms by which these viruses contaminate alfalfa seeds remain uncharacterized. While viruses may enter maternal seed parts through infection of reproductive organs before embryogenesis [2], they can also invade the embryo after fertilization with infected pollen [1,2,3,4]. This study highlights the significant role that pollen infection may play in the vertical transmission of plant viruses in alfalfa.

## 2. Materials and Methods

### 2.1. Plant Material

Pollen was collected from 15 different alfalfa genotypes previously selected in an in-house pre-breeding program for disease resistance and good agronomic performance (Table 1).

**Figure 1:**
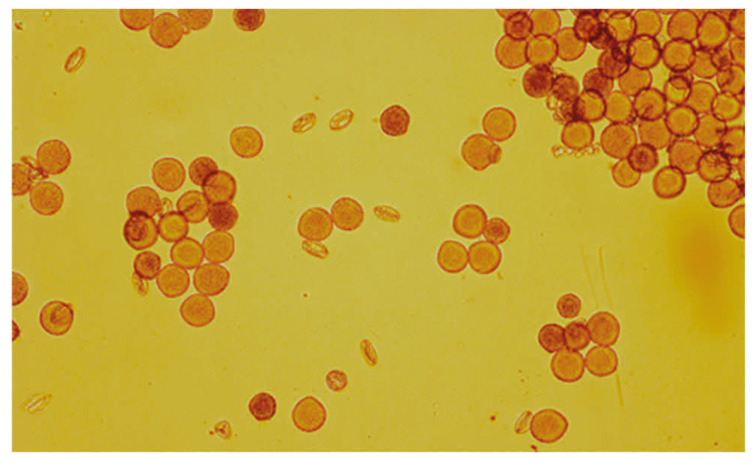
Clean alfalfa pollen samples obtained by manually tripping individual flowers and screened for vegetative debris using a Leica DM2000 microscope at 40× (Leica Microsystems, Buffalo Grove, IL, USA).

Plants were carried over from the 2024 growing season specifically for pollen collection from previous year polycrosses. Original plant germplasm came from the USDA-ARS National Plant Germplasm System alfalfa collection. Once regrowth began in the early summer, alfalfa plants were covered by a 6.1 × 6.1 × 1.8 m insect-proof cages for exclusion of potential pollinators. The field site was on Washington State University’s Irrigated Agriculture Research and Extension Center, where the USDA-ARS Plant Germplasm Introduction and Testing Research Unit (PGITRU) has a worksite. Branches with racemes and many recently opened flowers were collected from four clonally propagated plants of each of the 15 genotypes. These flower ‘bouquets’ were then transferred to the laboratory, kept turgid with base in water and used to collect pollen into 1.5 mL microfuge tubes (Eppendorf North America Inc., Enfield, CT, USA). On average, approximately 750 flowers per genotype were used for pollen collection. Individual flowers were tripped with a 1 mL pipette tip cut at an angle (Figure S1). The methodology yielded pure pollen samples, free of visible contamination from vegetative tissues (Figure 1).

At regular intervals (e.g., every 50–100 flowers), pollen at the end of the pipette tip was transferred with a pipettor to 1 mL of Trizol Reagent (Thermo Fisher Scientific Inc., Waltham, MA, USA) by aspirating and expelling liquid a few times. Samples were briefly stored at 4 °C before RNA extraction (Figure S2). Several methods were tested for pollen storage and preservation after collection and prior to the RNA extraction, including the use of RNAlater and TRIzol reagents, both supplied by ThermoFisher Scientific (Waltham, MA, USA). Microscopic observations showed that in RNAlater, pollen grains looked wrinkled and shrunken after a day of storage, while pollen samples in TRIzol Reagent looked mostly intact and free of any visible contaminants (Figure 1 and Figure S3).

### 2.2. Total RNA Extraction, RNA Sequencing and RT-PCR

Before RNA extraction, pollen samples stored in TRIzol Reagent were disrupted using a FastPrep-24 5G homogenizer (MP Biomedicals, Irvine, CA, USA) to access viruses inside the pollen grains. Pollen was transferred to 2 mL FastPrep tubes containing lysing matrix D (MP Biomedicals, Irvine, CA, USA) and homogenized 5 times at maximum speed (10 m/s) for 1 min each time. Once the samples were examined under the microscope and found to be sufficiently lysed and disrupted, with their cytoplasm released (Figure S4), they were processed for total RNA extraction, following the manufacturer’s protocol for TRIzol Reagent. After completion, the final eluate was additionally processed through Qiagen’s RNeasy Plant Mini Kit for RNA Extraction (Qiagen Inc., Germantown, MD, USA). RNA libraries were prepared using the ribosomal RNA depletion method and were sequenced on an Illumina NovaSeq X platform (Illumina, San Diego, CA, USA) (PE150) by CD Genomics (Shirley, NY, USA). All 15 samples were sequenced in a dedicated single lane to avoid potential cross-contamination from different projects. The RNA sequencing depth was 6 Gb per sample. Reverse transcription–polymerase chain reactions (RT-PCRs) were performed using the Titan One Tube RT-PCR System according to the manufacturer’s directions (Roche Diagnostics, Mannheim, Germany). Primers specific to each tested virus were designed based on the results of the HTS and are shown in Table S1. To ensure a complete absence of viral contamination (if used as controls, “healthy” alfalfa tissues may contain a variety of viral infections [14]), sterile RNAse-free water was used in control RT-PCR reactions along with additional no RT reaction controls as recommended by the manufacturer. The RT-PCRs were carried out in two technical replications.

### 2.3. Bioinformatics Analysis

Bioinformatics analyses were performed as previously described [13,15]. Briefly, sequence reads were trimmed using Trimmomatic (v0.40) [16] and then assembled with SPAdes (v3.15.5) [17]. The resulting contigs were screened using BLASTx (v2.15.0+) searches [18] against a virus database containing all plant virus protein sequences from the NCBI RefSeq database (https://www.ncbi.nlm.nih.gov/refseq/, accessed on 20 August 2025). The resulting potential plant viral hits were searched once again using BLASTx against the full NCBI nr protein database. BBMap (v39.01) [19] was used to generate sequencing coverage values for the final hits. Insect contamination in the pollen was estimated with Kraken2 (v2.1.3) [20] using the core-nt database to classify the trimmed HTS read pairs (https://benlangmead.github.io/aws-indexes/k2, accessed on 24 November 2025). Samples averaged 93.9% classified reads.

## 3. Results and Discussion

**Figure 2:**
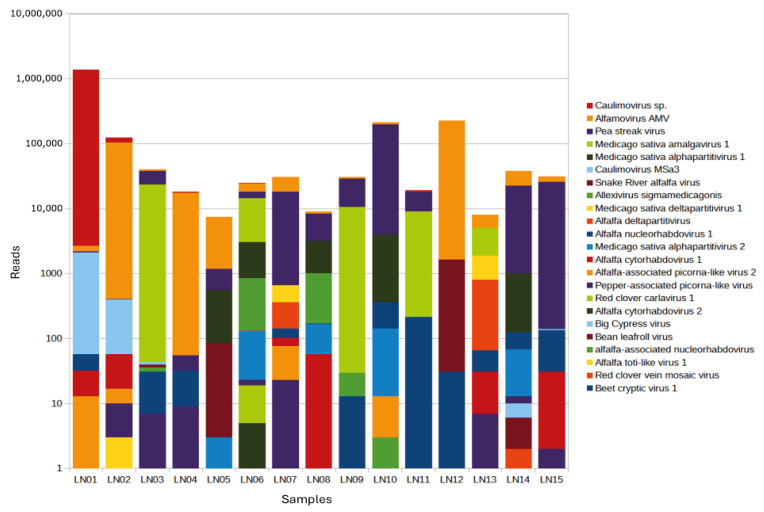
Distribution of viral communities among alfalfa pollen samples collected from 15 different genotypes.

In total, sequencing reads belonging to 22 viruses were found across all pollen samples collected from 15 different genotypes (Figure 2; Table 2 and Table S2). Of these 22 viruses, 14 were previously detected in alfalfa seeds [13]. Predictably, these viruses included AMV, partiti and amalga viruses, SRAV, and alfalfa virus S (AVS). While AMV is known to be pollen- and seed-transmitted, experimental evidence for pollen contamination was lacking for members of the *Partitiviridae* and *Amalgoviridae* families, AVS (*Alphaflexiviridae*) and the taxonomically unclassified SRAV, even though their seed transmissibility suggests that pollen infection may occur [13,21].

More compelling is that reads of bean leafroll virus (BLRV) and pea streak virus (PeSV), which traditionally are not considered seedborne viruses, were identified in one sample and all 15 samples, respectively (Table 2 and Table S2). This study marks the third instance of detecting these two viruses in alfalfa reproductive germplasm [13,21]. While both viruses are currently categorized as minor pathogens in alfalfa, they are aphid-transmitted and pose a significant threat to other economically important legume crops [22].

Consistent detection of cyto- and nucleorhabdoviral sequencing reads in alfalfa seeds, vegetative tissues and now in pollen samples, may indicate that they potentially represent endogenous viral elements (EVEs) integrated into the plant’s genome [23]. The integrated nucleocapsid protein genes of cytorhabdoviruses were previously found in the genomes of nine plant families, although the mechanism by which the viral RNA sequences were converted to DNA and incorporated into plant genomes remains unknown [24,25]. Hypothetically, the integration could be facilitated by reverse transcriptase encoded by other elements, such as pararetroviruses or retrotransposons [24]. Pararetroviruses of the *Caulimoviridae* family, *Caulimovirus* spp., were identified in all 15 samples (Table 2). The EVEs of two dsDNA reverse-transcribing plant pararetroviruses of this family were previously found to be stable constituents of the alfalfa genome [23].

Equally interesting is the identification of the red clover vein mosaic virus (RCVMV), a member of the genus *Carlavirus* (family *Betaflexiviridae*), in the alfalfa pollen samples. While RCVMV is known to infect alfalfa [26,27], to our knowledge, its vertical transmission in the crop has not been reported.

In alfalfa, RCVMV can cause reduced forage biomass and quality through symptoms like crinkling, interveinal mosaic, small leaves, yellowing, stunting, and mottling [26]. In alfalfa breeding programs for developing winter hardiness, RCVMV caused premature death of some breeding lines [22]. Alfalfa can also be a reservoir for RCVMV, leading to the spread of the virus to other important legume crops that are also susceptible to this pathogen [22].

One of the pollen samples contained sequencing reads of beet cryptic virus (BCV), the first discovered member of the family *Partitiviridae* to infect alfalfa [28]. Biological effects of BCV on alfalfa are unclear, although in sugar beet, BCV infection was associated with significantly reduced root and sugar yields [29].

Sequencing reads with high-scoring matches (>99% nucleotide identity) to the pepper-associated picorna-like virus (PAPLV) were identified in eight alfalfa pollen samples. Except for the nucleotide sequence (PP728253.1), we could not find any information on the biology or phylogeny of PAPLV. Although pepper plants in Korea are infected by a variety of viruses including economically important picorna-like viruses [30], PAPLV has not been documented among them.

Several other viral sequences found in pollen could have originated from contamination by arthropods (big Cypress virus and alfalfa-associated picorna-like virus 2) or fungal and protozoan hosts (alfalfa toti-like virus 1). Importantly, the collection protocol employed in this study effectively eliminated the risk of contamination from vegetative tissues (see Section 2.1.), and the only potential contaminants likely originated from insects feeding on alfalfa flowers, or from fungal and protozoan hosts carried by pollinators.

Although aphids are the primary insect vectors for AMV, BLRV, PeSV, and RCVMV, all of which were detected in pollen samples, they do not feed on pollen and mostly congregate on stems and leaves. This was evident in the very low count of sequencing reads associated with pea aphid (*Acyrthosiphon pisum*), one of the most efficient vectors of AMV, BLRV, PeSV and RCVMV (on average, 1.34874 × 10^−6^ of the total reads per sample; Table S3). Nevertheless, the possibility that BLRV, PeSV, and RCVMV sequences originated from trace aphid debris or microscopic body parts cannot be entirely excluded. This is particularly relevant for BLRV and RCVMV, both of which were associated with a limited number of samples and a low abundance of sequencing reads (Table S2). The same could possibly apply to SRAV as the virus was previously reported in Western flower thrips (*Frankliniella occidentalis*) feeding on alfalfa stands [31], and a limited number of *F. occidentalis* reads were identified in the pollen samples (on average, 0.0001 of the total reads per sample; Table S3). However, given that our previous results established the presence of SRAV in surface-sterilized alfalfa seeds and seedlings germinated from them, we believe that the detection of the virus in the pollen grains is authentic.

On the contrary, PeSV reads were identified in each of the 15 tested samples, which likely suggests a close association with the pollen grain, either on its surface or within. Ultimately, whether the viruses were housed within insect debris, a small amount of which could pollute the pollen samples, or associated directly with the pollen grains may be irrelevant. In either scenario, virus-carrying insects contaminating pollen samples could easily be dispersed concomitantly, thus causing viruses to spread throughout the crop.

**Figure 3:**
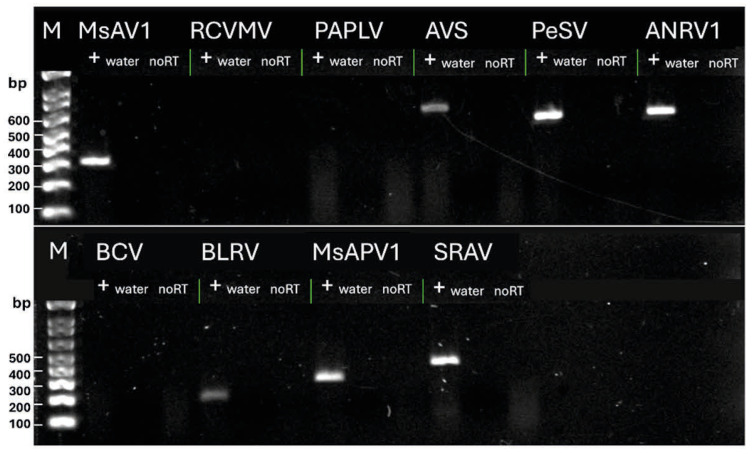
Reverse transcription–polymerase chain reaction (RT-PCR) was employed to verify the presence of a representative subset of viral sequences identified in the alfalfa pollen samples. “+” indicates RT-PCR product obtained from a pollen sample; “water” indicates a control reaction with sterile RNAse-free water added instead of a pollen sample; “no RT” indicates a control reaction with all components present except the RT enzyme mix. M, 1 kb DNA ladder (Thermo Fisher Scientific Inc., Waltham, MA, USA); Medicago sativa amalgavirus 1 (MsAV1); RCVMV, red clover vein mosaic virus; PAPLV, pepper-associated picorna-like virus; AVS, alfalfa virus S; PeSV, pea streak virus; ANRV, alfalfa nucleorhabdovirus; BCV, beet cryptic virus; BLRV, bean leafroll virus; MsAPV, Medicago sativa alphapartitivirus; SRAV, Snake River alfalfa virus.

We used reverse transcription–polymerase chain reactions to confirm the presence of several arbitrarily chosen viruses that were initially identified by sequencing in alfalfa pollen samples (Figure 3). The RT-PCR assays were conducted on samples identified as positive for each respective virus via HTS. Primers for RT-PCRs were designed based on the obtained HTS contigs (Table S1). The viruses included AVS, BLRV, PeSV, SRAV, PAPLV, BCV, RCVMV, ANRV 1, Medicago sativa amalgavirus 1 (MsAV1), and Medicago sativa alphapartitivirus 1 (MsAPV1). The RT-PCR products were successfully amplified from samples that tested positive for every virus via HTS, excluding PAPLV, RCVMV, and BCV (Figure 3).

It is plausible that the quantity of viral genetic material for the latter three viruses fell below the threshold of reliable detection via RT-PCR, given the limited number of sequencing reads observed in the HTS data (Table S2). Consequently, the low viral load may be insufficient to initiate infection, thus questioning the epidemiological significance of these viruses.

## 4. Conclusions

Based on our current information, this is the first study of the alfalfa pollen virome carried out by HTS technology. As of today, the experimental knowledge on alfalfa pollen infection with plant viruses was limited to AMV [11,12]. In this work, in addition to AMV, we identified sequencing reads belonging to several other viruses infecting and/or contaminating alfalfa pollen grains, including partiti and amalga viruses, SRAV, AVS, BLRV, PeSV, RCVMV, PAPLV, and other species. We believe SRAV, AVS, BLVR, and PeSV are strong candidates for further research into their putative natural transmissibility through alfalfa pollen.

The presence of the arbitrarily chosen viruses (SRAV, AVS, BLRV, PeSV, ANRV, MsAPV1, and MsAV1) in alfalfa pollen samples was independently validated via RT-PCR with virus-specific primers. These findings are consistent with earlier HTS-based characterization of the alfalfa seed virome [13] and highlight pollen infection as a potential pathway for vertical virus transmission.

Vertical transmission facilitates the early introduction of a virus into a host population, increasing the risk of widespread dissemination and subsequent epidemics [2,32]. Notably, alfalfa’s role as a viral reservoir creating a persistent source of infection poses a considerable threat that may lead to substantial losses in other crops [22]. Finally, since virus infections are known to negatively affect reproductive characteristics of the pollen grains and their overall yield [33,34,35], it is anticipated that their association with alfalfa pollen may further reduce its performance, thus possibly impacting seed production.

The practical implications of this research are also relevant to the specific tested genotypes, originally sourced from the USDA-ARS National Plant Germplasm System (NPGS). The selected genotypes are currently being used in pre-breeding efforts to improve alfalfa for fungal disease resistance and good agronomic performance. Finding viral plant pathogens associated with the pollen, including some with reported vertical transmission, is important information to consider as improved selections progress.

## Figures and Tables

**Table 1:** Alfalfa (*Medicago sativa* L.) plant genotype information used for sourcing pollen from field-grown plants in Prosser, WA, USA.

No.	Accession *	Name	Origin	Improvement Level
1	PI 517233	ILCA 5674	Syria	Wild material
2	PI 552545	UC 222	United States	Breeding material
3	PI 516870	Demnate	Morocco	Landrace
4	PI 536526	MNGRN-2	United States	Cultivar
5	PI 251560	No. 1	Former Serbia & Montenegro	Cultivated material
6	PI 516796	GR 643	Morocco	Landrace
7	PI 233199	G 13621	Russian Federation	Uncertain status
8	PI 399535	Hunter River	Australia	Cultivar
9	PI 632163	P.F. 5866	Chile	Cultivated material
10	PI 231765	G 5184	United States	Uncertain status
11	PI 251693	No. 22503	Russian Federation	Uncertain status
12	PI 231042	No. 4	India	Uncertain status
13	PI 320535	Crioula	Brazil	Landrace
14	PI 233195	G 13620	Russian Federation	Uncertain status
15	PI 255962	Rambler	Canada	Cultivar

* Individual plants (genotypes) were selected in pre-breeding efforts from the plant introduction (PI) accessions which were originally obtained from the USDA ARS National Plant Germplasm System (NPGS).

**Table 2:** A list of viruses identified by HTS in alfalfa pollen collected from 15 different germplasm sources.

Virus Names	Proposed Taxonomy	No. Alfalfa Genotypes *
1. Alfalfa cytorhabdovirus 1	*Rhabdoviridae*, *Cytorhabdovirus*	7
2. Alfalfa cytorhabdovirus 2	*Rhabdoviridae*, *Cytorhabdovirus*	1
3. Alfalfa deltapartitivirus	*Partitiviridae*, *Deltaparitivirus*	3
4. Alfalfa nucleorhabdovirus 1	*Rhabdoviridae*, *Betanucleorhabdovirus*	12
5. Alfalfa toti-like virus 1	*Orthototiviridae*, unclassified	1
6. Alfalfa-associated picorna-like virus 2	*Picornavirales*, *Iflaviridae*, *Iflavirus*	4
7. Alfamovirus AMV	*Bromoviridae*, *Alfamovirus*	15
8. Allexivirus sigmamedicagonis (alfalfa virus S)	*Alphaflexiviridae*, *Allexivirus*	4
9. Bean leafroll virus	*Tombusviridae*, *Luteovirus*	1
10. Beet cryptic virus 1	*Partitiviridae*, *Alphacryptovirus*	1
11. Big Cypress virus	*Orbivirus*	1
12. Caulimovirus MSa3	*Caulimoviridae*, unclassified	5
13. Caulimovirus spp.	*Caulimoviridae*, *Caulimovirus*	15
14. Medicago sativa alphapartitivirus 1	*Partitiviridae*, *Alphapartitivirus*	5
15. Medicago sativa alphapartitivirus 2	*Partitiviridae*, *Alphapartitivirus*	5
16. Medicago sativa amalgavirus 1	*Amalgaviridae*, unclassified	5
17. Medicago sativa deltapartitivirus 1	*Partitiviridae*, *Deltaparitivirus*	2
18. Pea streak virus	*Betaflexiviridae*, *Carlavirus*	15
19. Pepper-associated picorna-like virus	*Picornavirales*, unclassified	8
20. Red clover vein mosaic virus	*Betaflexiviridae*, *Carlavirus*	2
21. Snake River alfalfa virus	unclassified	3
22. Alfalfa-associated nucleorhabdovirus	*Rhabdoviridae*, *Betanucleorhabdovirus*	1

* The number of alfalfa genotypes in which individual viruses were identified.

## Data Availability

All sequences mentioned in the text are included in the Supplementary Materials (Table S2). Raw data are available in the NCBI SRA under the BioProject ID: PRJNA1434363.

## Supplementary Materials

The following supporting information can be downloaded at https://www.mdpi.com/article/10.3390/v18040408/s1: Figure S1. Individual flowers were tripped with a 1 mL pipette tip cut at an angle for pollen collection. Figure S2. Individual pollen samples were stored in Trizol Reagent (Thermo Fisher Scientific Inc., Waltham, MA, USA). Figure S3. Comparison between pollen samples stored in RNAlater (A) and TRIzol reagents (B) (both supplied by Thermo Fisher Scientific, Waltham, MA, USA); Olympus BX53, 40×. Figure S4. Alfalfa pollen samples disrupted in FastPrep-24 5G homogenizer (MP Biomedicals, Irvine, CA, USA); Olympus BX53, 40×. Table S1. Virus-specific primers designed based on the HTS data. Table S2. Viral contigs, sequences, BLAST hits, coverage, and descriptions. Table S3. Identification of *Acyrthosiphon pisum* and *Frankiniella occidentalis* sequencing reads in alfalfa pollen samples.

## Abbreviations

AVS	alfalfa virus S
ACRV	alfalfa cytorhabdovirus
ANRV	alfalfa nucleorhabdovirus
BCV	beet cryptic virus
BLRV	bean leafroll virus
HTS	high-throughput sequencing
MsAV	Medicago sativa amalgavirus
MsAPV	Medicago sativa alphapartitivirus
NPGS	National Plant Germplasm System
PAPLV	pepper-associated picorna-like virus
PeSV	pea streak virus
RCVMV	red clover vein mosaic virus
SRAV	Snake River alfalfa virus
RT-PCR	reverse transcription–polymerase chain reaction
