# Supplementary material for: Characterization of the Alfalfa Pollen Virome
Source: Viruses. 2026 Mar 25;18(4):408. doi: 10.3390/v18040408 (PMC13119625; doi:10.3390/v18040408)
Supplement: Supplementary file 1 [file viruses-18-00408-s001.zip › viruses-4188316-supplementary.pdf]

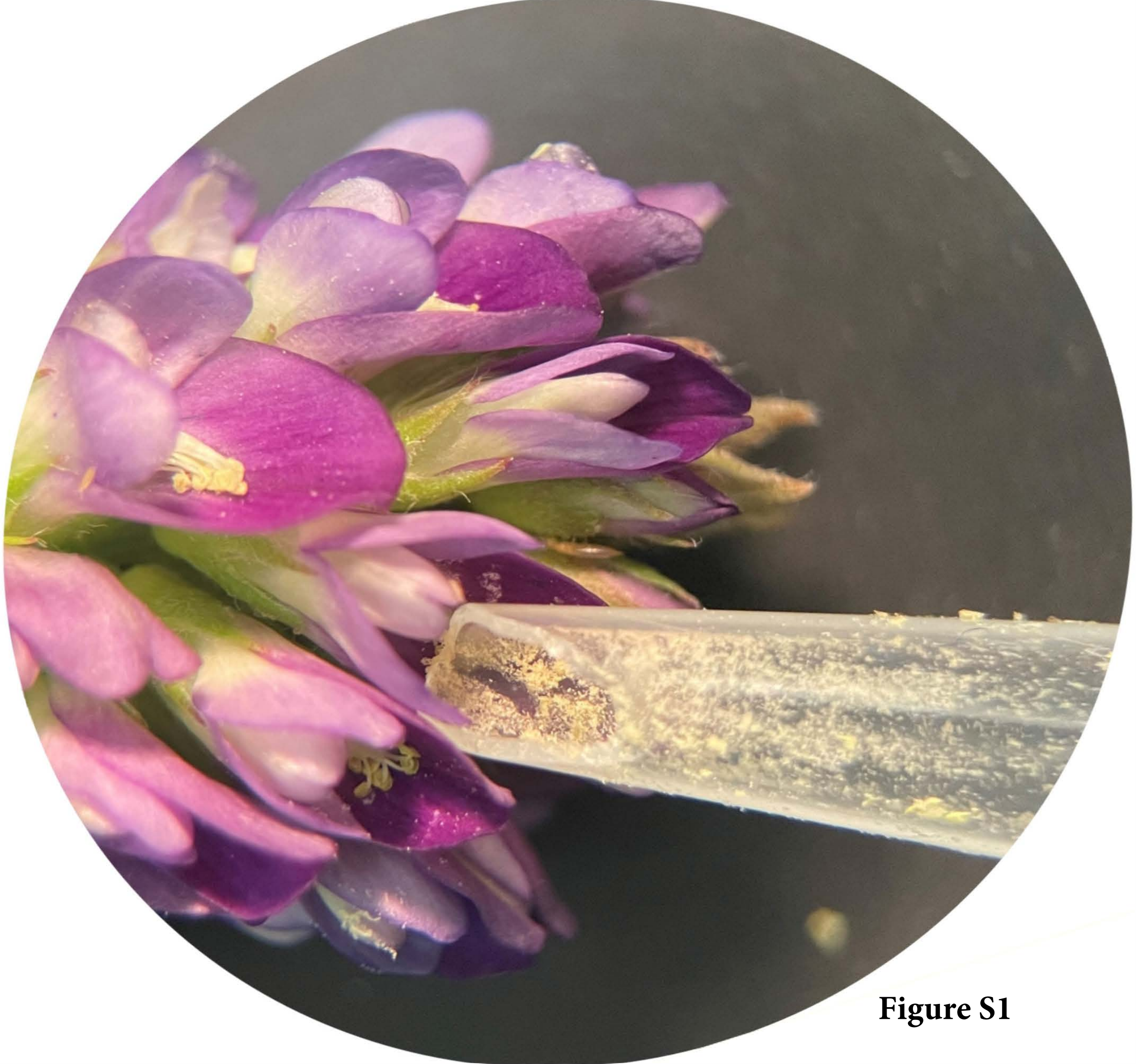

Figure S1

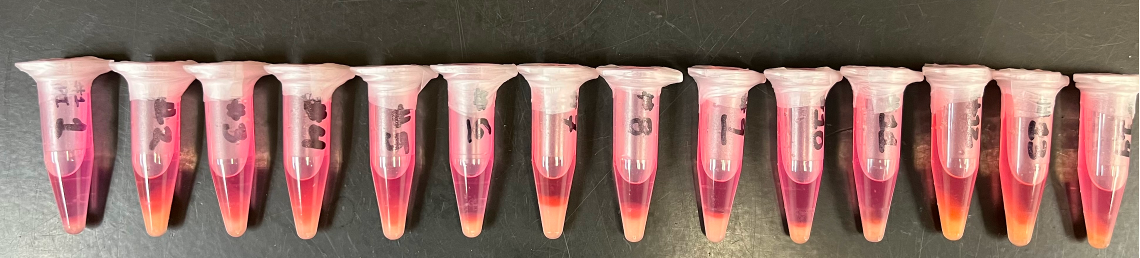

Figure S2

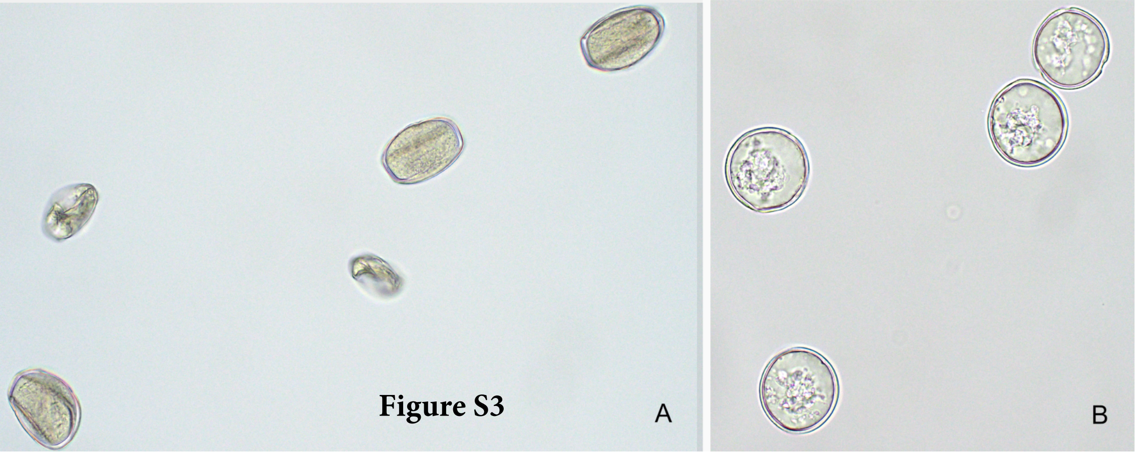

Figure S3

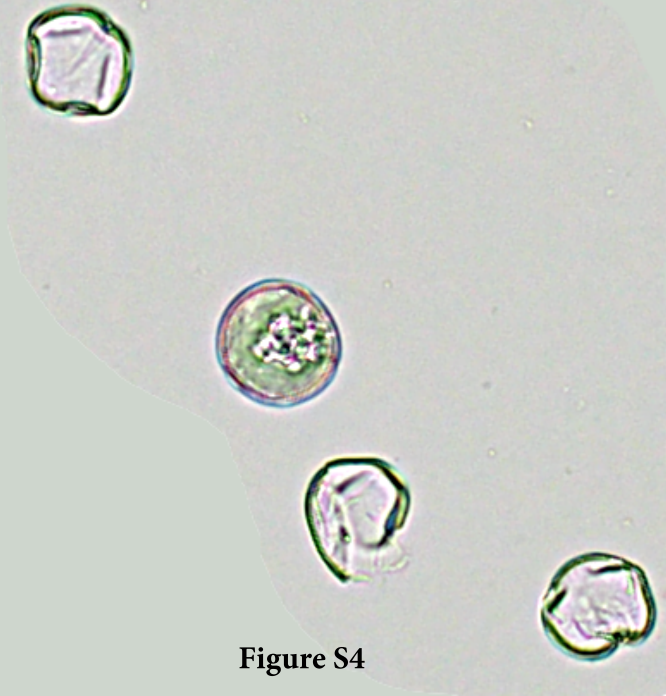

Figure S4

Table S1

Virus-specific primers designed based on the HTS data

Name	Sequence	Product length
PAPLV-F	TGGGAGAAGTAGAAGAGGAAGTA	
PAPLV-R	CCAGGCACACCTGATGTATAA	314
RCVMC-F	TGCTGTAGTAAGTGTGGTAAGTG	
RCVMC-R	GGTCATACCTTAAGCACCAAGA	210
BCV-F	CGTAGCCGTAAGAGGCTTG	
BCV-R	AGAACATTGGTGAAAGTGAGGA	207
BLRV-F	GAGATGCTATTGTTAAAGATGTTGGATG	
BLRV-R	AGAACATTGGTGAAAGTGAGGA	205
PeSV-F	GCCTCACGTTGGAGACAATA	
PeSV-R	CCCTCAGCATCCCGAATAAA	554
AVS-F	GGCCTTTACCAAGACGGTAATA	
AVS-R	GTAGCGGTGATTGTTGGATTTG	639
SRAV-F	GAGTCGTTGGCTAAGGTGATAC	
SRAV-R	GCCGCTAAACCTACTCCTATTC	407
ANRV-F	CCCACCTAAGCCACCTTTAAT	
ANRV-R	CAGAGCTACAGAGGGTGATTTC	576
MsAV1-F	AGTGTGGAGGACCCATTTATTC	
MsAV1-R	GGGTGGTGATGTAGCCAATTA	317
MsAPV1-F	GGATGAACTCGACCCTAAGAAC	
MsAPV1-R	CAAGCCCGACGAAAGTAGAA	303

Table S3

Identification of *Acyrthosiphon pisum* and *Frankiniella occidentalis* sequencing reads in alfalfa pollen samples.

Samples	[1]*A. pisum* reads	[2]*F. occidentalis* reads	Total sample reads
LN01	75	16131	81613114
LN02	90	1844	75906416
LN03	55	1330	86806392
LN04	50	6172	66130554
LN05	282	1322	82527766
LN06	80	175	68517156
LN07	36	738	63695260
LN08	86	9881	72679330
LN09	97	195	83426152
LN10	103	12345	72358236
LN11	218	27929	88360804
LN12	93	351	100416738
LN13	42	5911	56290254
LN14	129	29934	57653144
LN15	85	10743	71337634
Average	101.4	8333.4	75181263.33
Part/total	1.34874E-06	0.000110844	
1, *Acyrthosiphon pisum*			
2, *Frankliniella occidentalis*			
